# Predictive and Prognostic Roles of Gut Microbial Variation in Liver Transplant

**DOI:** 10.3389/fmed.2022.873523

**Published:** 2022-05-10

**Authors:** Hon Jen Wong, Wen Hui Lim, Cheng Han Ng, Darren Jun Hao Tan, Glenn K. Bonney, Alfred W. C. Kow, Daniel Q. Huang, Mohammad Shadab Siddiqui, Mazen Noureddin, Nicholas Syn, Mark D. Muthiah

**Affiliations:** ^1^Division of Gastroenterology and Hepatology, Department of Medicine, National University Health System, Singapore, Singapore; ^2^Yong Loo Lin School of Medicine, National University Singapore, Singapore, Singapore; ^3^National University Centre for Organ Transplantation, National University Health System, Singapore, Singapore; ^4^Division of Hepatobiliary and Pancreatic Surgery, Department of Surgery, National University Hospital Singapore, Singapore, Singapore; ^5^Division of Gastroenterology, Hepatology and Nutrition, Department of Internal Medicine, Virginia Commonwealth University, Richmond, VA, United States; ^6^Cedars-Sinai Fatty Liver Program, Division of Digestive and Liver Diseases, Department of Medicine, Comprehensive Transplant Centre, Cedars-Sinai Medical Centre, Los Angeles, CA, United States

**Keywords:** liver transplantation, gut microbiome, immunity, rejection, cancer, metabolic disease, prevention

## Abstract

Patients undergoing liver transplant (LTX) typically confront a challenging postoperative journey. A dysbiotic gut microbiome is associated with the development of complications, including post-LTX allograft rejection, metabolic diseases and *de novo* or recurrent cancer. A major explanation of this are the bipartite interactions between the gut microbiota and host immunity, which modulates the alloimmune response towards the liver allograft. Furthermore, bacterial translocation from dysbiosis causes pathogenic changes in the concentrations of microbial metabolites like lipopolysaccharides, short-chain fatty acids (SCFAs) and Trimethylamine-N-Oxide, with links to cardiovascular disease development and diabetes mellitus. Gut dysbiosis also disrupts bile acid metabolism, with implications for various post-LTX metabolic diseases. Certain taxonomy of microbiota such as lactobacilli, *F.prausnitzii* and *Bacteroides* appear to be associated with these undesired outcomes. As such, an interesting but as yet unproven hypothesis exists as to whether induction of a “beneficial” composition of gut microbiota may improve prognosis in LTX patients. Additionally, there are roles of the microbiome as predictive and prognostic indicators for clinicians in improving patient care. Hence, the gut microbiome represents an exceptionally exciting avenue for developing novel prognostic, predictive and therapeutic applications.

## Introduction

Liver transplant (LTX), the only treatment for patients with end-stage liver disease, liver failure and hepatocellular carcinoma (HCC) with a definite long-term survival benefit, experiences relatively high incident rates for postoperative complications (1). Hence, we sought to review how variations in gut microbiota post LTX influences the incidence of rejection, metabolic disease and cancers in the liver to better understand and prevent such complications.

A complex and diverse population of microorganisms, known as the gut microbiome, exists in the human gastrointestinal tract. Through dynamic microbiota-microbiota and microbiota-host interactions, involving a myriad of by-products, the gut microbiome contributes beneficial or pathological influences on host health such as in maintaining metabolic homeostasis, intestinal integrity, and regulating the host immune system.

**Figure 1:**
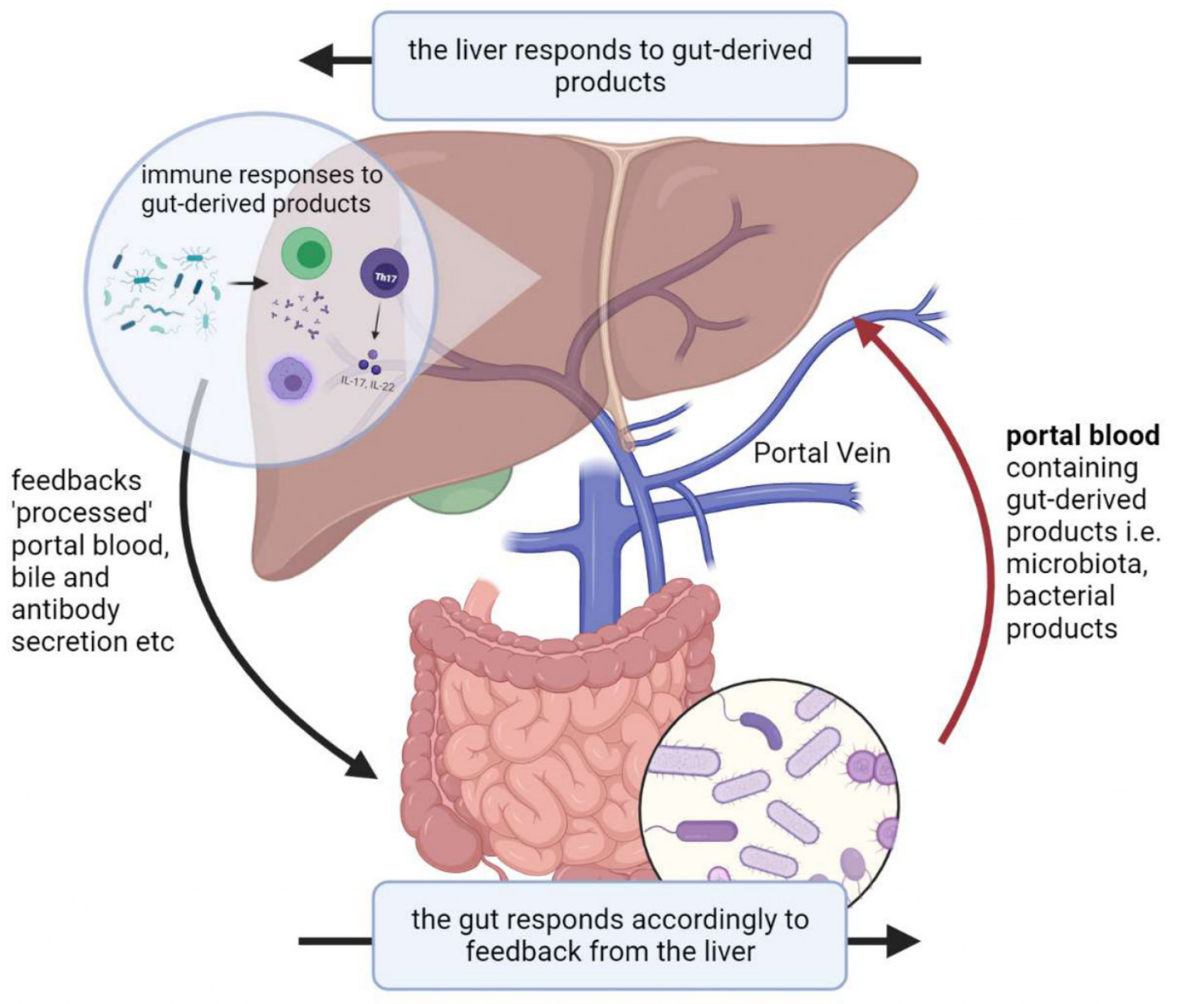
Illustration of the gut-liver axis.

Given its anatomical position, the liver exhibits a bidirectional relationship with the gut and its microbiota, known as the gut-liver axis which exhibits circular causality. The liver thus represents the first line of defense against gut-derived antigens and toxicity factors (2). The liver faces increased pressure during gut microbial dysbiosis, which is defined as any change to the composition of resident commensal microbiota relative to the microbiome found in healthy individuals (3). This increased pressure partly arises from its associations with intestinal epithelial barrier dysfunction which increases exposure of the liver to bacterial components *via* the gut-liver axis, resulting in hepatic injury (Figure 1). In part, dysbiosis is implicated in the pathogenesis of various liver diseases such as liver cirrhosis, HCC, non-alcoholic fatty liver disease, and other metabolic changes (4).

LTX often corresponds with changes in the composition of the gut microbiome. Such changes can occur due to altered anatomy from surgery, biliary complications post-LTX affecting bile acid secretion into the intestines, use of immunosuppression and antibiotics. Reasons include introduction of donor microbiota to the recipient *via* the liver allograft. Surgery temporarily increases intestinal permeability, allowing certain opportunistic pathogens to enter the portal or systemic circulation (5–7). Allografts carry donor immune cells that can interact with recipient gut microbiome *via* the gut-liver axis, resulting in changes in microbial composition. An example of such changes in the gut microbiome of post-LTX patients can include a decrease in *Bifidobacterium, Lactobacillus, Faecalibacterum prausnitzii* and an increase in *Enterobacteriaceae, Enterococcus* during a qPCR-based analysis of 111 LTX patients (8).

## Post-LTX Gut Microbial Variation

### Immunology

#### Tolerance of the Liver Allograft

In general, regulatory T cells (Tregs) contribute toward the tolerative nature of the liver allograft by influencing the development and maintenance of immunological tolerance to self and alloantigens. For example, Tregs inhibit effector T cells by secreting inhibitory cytokines and interacting with CD80/CD86 through immune checkpoint CTLA-4, which downregulates T cell activation (9). Hence, Tregs help prevent the development of acute cellular rejection (ACR) by inducing a tolerogenic environment in LTX patients with an intact immune system.

Post-LTX dysbiosis causes abnormal increases in portal circulation of bacterial products like LPS. Kupffer cells, resident liver macrophages participating in initial immune responses to pathological challenges, respond by increasing concentrations of the anti-inflammatory cytokine IL-10 in the liver (10). Additionally, translocation of gut microbiota induces type 1 interferon and stimulates myeloid cell IL-10 production, thereby increasing hepatic IL-10 concentrations (11). Since Treg expression is dependent on IL-10 signaling (12), alloreactive T cell proliferation is further suppressed, thus promoting allograft tolerance.

LPS-induced local inflammation upregulates costimulatory molecule CD80 in a murine model (13). In humans, CD80 signaling regulates apoptosis and inhibits responses of activated CD8+ T cells *via* immune checkpoint PD-L1 (14). If the findings of the murine model are applicable to humans, upregulated CD80 from post-LTX dysbiosis can increase apoptosis of CD8+ T cells and inhibit alloimmune responses.

With regards to the specific changes in the composition of the gut microbiome, an increase in gut *Bacteroides fragilis* and *Bacteroides thetaiotaomicron* which drive Treg induction and differentiation in post-LTX dysbiosis (15) was found to be correlated with a more tolerant alloimmune response.

Nevertheless, a potential over-suppression of the immune status due to post-LTX dysbiosis, for example, due to excessive upregulation of Tregs from immoderate concentrations of IL-10 that leads to reduced alloreactive T cell proliferation, will increase risk of infections and cancer. This can result in increased mortality rates in LTX patients.

#### Rejection of the Liver Allograft

Adaptive immune responses *via* mesenteric lymph nodes, a key immunological component of the gut is transformed by commensal gut bacteria, primarily by stimulating the differentiation of naïve T cells into Tregs (16). This suggests that pre-existing or LTX-induced dysbiosis involving a change in commensal gut bacteria can disrupt the balance of CD4+ T cell subsets in mesenteric lymph nodes. Migration of these altered mesenteric lymph node CD4+ T cells into the liver allograft can promote hepatic injury (17) and accelerate the pathogenesis of early ACR.

Post-LTX hepatic ischemia-reperfusion (I/R) injury is correlated with increased IL-17 levels in portal vein plasma and the small intestine (18). A murine model demonstrated how increased levels of IL-17 significantly suppresses Treg expansion, hence increasing alloreactive T cell action on the allograft (19). This I/R injury-mediated process is hypothesized to occur relatively similarly and accelerate the development of early ACR in humans after LTX. Post-LTX dysbiosis can involve increases in segmented filamentous bacteria, which aid in IL-17 expression (19), alongside increases in *lactobacilli*. Increased *lactobacilli* upregulate IL-17 through Peyer's patches' resident T lymphocytes based on another murine model (20). Hence, it is possible for dysbiosis to exacerbate I/R injury-mediated development of early ACR in the human liver if similar interactions occur.

In contrast, the gut microbiome can protect against hepatic I/R injury-associated rejection. For instance, hepatic I/R injury induces Paneth cell degranulation and inhibits their immune response. This is implicated in the worsening of liver injury (18). Degranulation of mast cells during hepatic I/R injury has strong positive correlation to liver damage (21), which deteriorates allograft function. However, alterations in gut microbiota involving butyrate have the potential to improve I/R injury-induced hepatocyte injury by preventing NF-kB activation, upregulate the gut microbial metabolite, 3,4-dihydroxyphenylpropionic acid, which mitigates macrophage pro-inflammatory activity and stimulate the protective effect of nucleotide-binding oligomerization domain-containing protein 2 (NOD2) signaling by gut microbiota, has shown to alleviate hepatic I/R injury (22–24). A study conducted by Ito et al. indicated how increased severity of hepatic I/R injury corresponded to markedly poorer outcomes relating to early allograft dysfunction (25). As such, an altered gut microbiome after LTX that leads to alleviated hepatic I/R injury may be able to play a role in improving early ACR outcomes.

Increased bacterial translocation from the gut into the liver allograft during dysbiosis increases antigen stimulation in the liver. Schurich et al. showed how low-dose antigen stimulation resulted in tolerance whilst high-dose antigen stimulation induced effector T cell differentiation in liver sinusoidal endothelial cell (LSEC) T cell presentation (26). This means increased differentiation of effector T cells, leading to an enhanced alloimmune response and thereby promoting ACR.

Specific cytokines have the potential to act as biomarkers or are involved in the risk of acute rejection after LTX. For example, elevated levels of intracellular cytokine IFN-γ and IL-2 expression in T cells are associated with an increased risk for the development of ACR (27). Dysbiosis influences the host immune response, in turn inducing abnormal production of inflammatory cytokines. In influencing the host immune response, dysbiosis is often associated with dysregulated production of inflammatory cytokines (28). This demonstrates a plausible role of a gut microbiome-based liver immunoassay in the prognoses of ACR, should the specific relationships between dysbiosis and cytokine concentrations be discovered in the future.

In addition, Ren et al. suggested the potential ability of intestinal microbiota variation in predicting early ACR after LTX using a murine model. They conducted real-time quantitative polymerase chain reaction tests which reflected decreased *F.prausnitzii* and *Lactobacillus*, whilst *Clostridium bolteae* increased during ACR. In example, if similar interactions occur in the liver, *F.prausnitzii* may be able to demonstrate beneficial effects on ACR, where *F.prausnitzii* endows dendritic cells with properties that promote Treg upregulation, for example *via* IL-10, alongside reduced effector T cell development in the human colon (29).

However, *F.prausnitzii* can metabolize the immunosuppressant tacrolimus into the M1 metabolite which is 15 times less potent in inhibiting T lymphocyte proliferation, thereby increasing the risk of developing ACR in patients requiring the use of tacrolimus for tolerance of the allograft (30). This highlights upon other potential interactions between gut microbiota and immunosuppressants which can affect the efficacy of post-LTX immunosuppression. Hence, it can be important for clinicians to have a greater understanding and awareness of microbiota-medication interactions to better treat post-LTX complications in their patients.

Persistent dysbiosis leads to persistent bacterial translocation into the liver allograft. This can lead to prolonged upregulation of inflammatory cytokines, which can enhance the development of chronic rejection, particularly if long-term inflammation is observed (31). Knowledge regarding the impact of the post-LTX microbiome on late ACR and CR is highly limited due to sparce research.

This section serves to highlight upon the therapeutic opportunities provided by the gut microbiome with regards to tolerance and rejection of the allograft. Although the liver is more tolerogenic than other organs, there can still be circumstances in which tolerance is not achieved. The ability to remove gut microbiome-related rejection factors is likely beneficial toward reducing overall risk of rejection and maintaining normal allograft function.

### Post-transplant Metabolic Disease

#### Post-transplant Diabetes Mellitus

The gut microbiome is responsible for the physiological homeostasis of bile acids (BAs), in which conjugated bile acids (CBAs) secreted into the duodenum are metabolized by gut microbiota. This involves the hydrolysis of CBAs into secondary bile acids, glycine and taurine *via* bile salt hydrolases (BSH) produced by intestinal bacteria, such as gram-positive species like *Lactobacillus*. BSH can carry out 7α-dehydroxylation of cholic acid and chenodeoxycholic acid to form deoxycholic acid and lithocholic acid (LCA). This involves a multistep biochemical pathway found only in anaerobic gut bacteria. BAs serve endocrinal functions mainly by agonizing Farnesoid X receptor (FXR) and G-protein coupled bile acid receptor TGR5. Translocation of bacteria during dysbiosis disrupts BA metabolism, leading to dysregulated agonism of FXR and TGR5. FXR enhances epithelial barrier integrity and plays a crucial role in hepatic triglyceride homeostasis (32). TGR5 improves glucose tolerance by contributing to improvements in hepatic insulin signaling (33). Hence, a lack of FXR and TGR5 signaling from a dysbiosis-mediated dysregulated BA pool can be implicated in the pathogenesis of insulin resistance and thus PTDM. This means that detection of BSH-related bacteria and pathogenic BA modifying bacteria in the postoperative period can aid in the prediction and prevention of PTDM incidence in the LTX patient.

Post-LTX bacterial translocation alongside dysbiosis can lead to low-grade constitutive increase in plasma LPS levels, thereby resulting in LPS-induced metabolic endotoxemia. A related study detected liver insulin resistance in LPS-infused mice (34), and hyperinsulinemia is correlated with the onset of diabetes. Increases in *Bacteroides* and *Prevotella* alongside decreases in *Firmicutes, Clostridia, and Bifidobacteria* (35) can be associated with the development of PTDM given the corresponding decrease in *Bifidobacteria* post-LTX (8).

**Figure 2:**
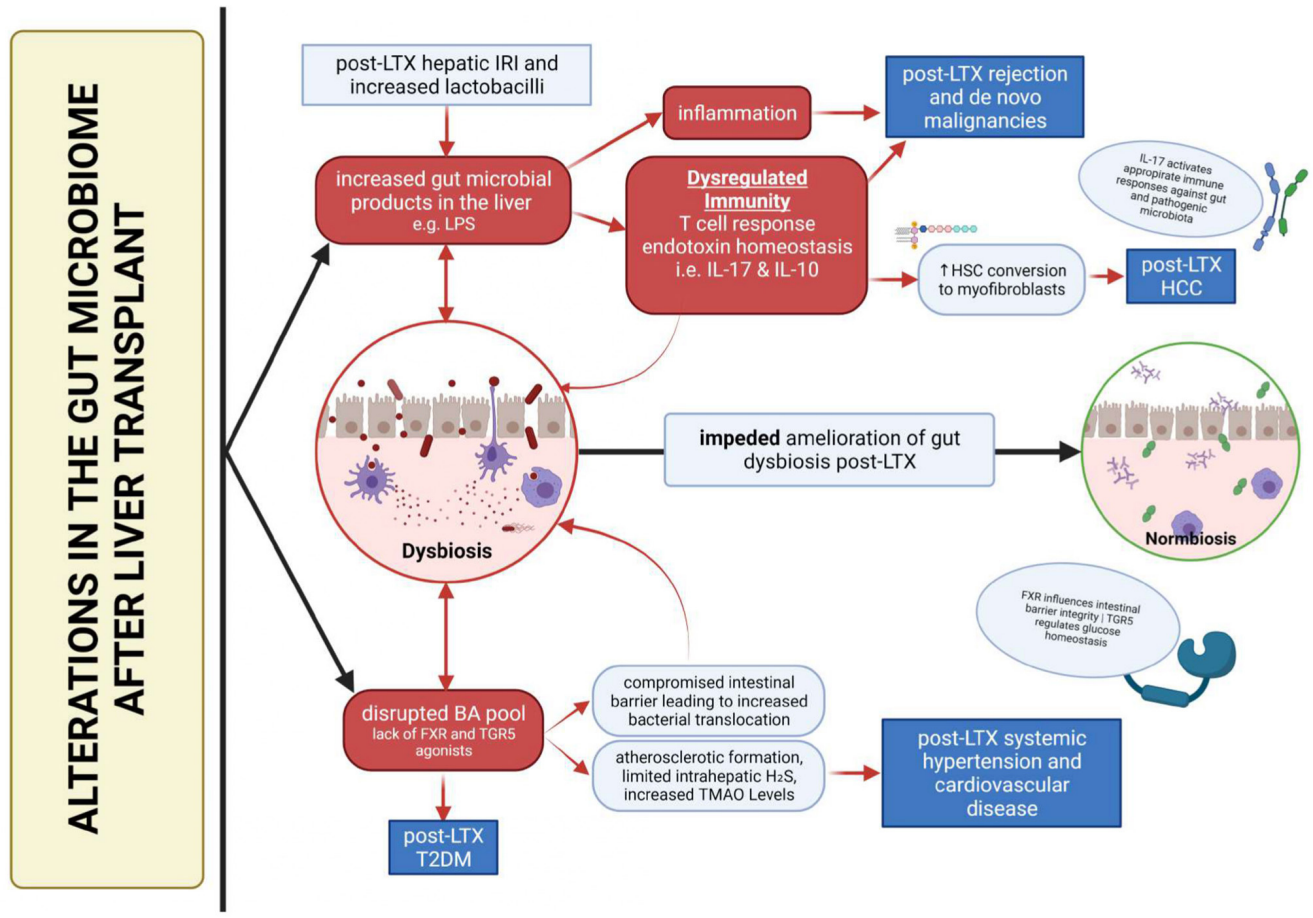
Summary of dysbiosis-mediated development of post-liver transplant complications.

On a separate note, the inevitable modification of the BA pool creates drastic consequences after LTX, particularly through the gut liver axis due to its antimicrobial nature. It allows increased colonization of the microbiome by pathogens, which in turn causes further disruption of the BA pool (36). BAs also modulate a wide range of pro and anti-inflammatory genes induced by LPS (37), a key player in influencing the development of post-LTX complications. This, in tandem with a dysregulated immunity, may result in the creation of a downward spiral, which prevents dysbiosis amelioration and exacerbates the development post-LTX complications (Figure 2).

#### Post-transplant Dyslipidemia

Dyslipidemia is characterized by abnormal blood lipid profiles. It includes abnormal levels of cholesterol, triglycerides, low-density lipoprotein (LDL) cholesterol, and high-density lipoprotein (HDL) cholesterol. Its etiology can include additional risk factors like obesity, hypertension and DM. For example, obesity has been associated with a decrease in gut microbiome diversity and elevated circulating LPS levels of patients with metabolic issues (38). Hyperinsulinemia from post-transplant and gut microbial factors leads to increased hepatic uptake of FFA, such that hepatic synthesis of very-low-density lipoprotein (VLDL) is increased (39). Additionally, insulin resistance leads to reduced lipoprotein lipase, which results in lower triglyceride clearance (40). Hence, there is increased VLDL to LDL cholesterol conversion, resulting in increased LDL cholesterol levels and hyperlipidemia.

### Post-transplant Systemic Hypertension and Cardiovascular Disease

Accumulating evidence points toward alterations in the gut microbiome playing a role in cardiovascular diseases (CVD). Hypertension (HTN), an established risk factor for CVD was found in patients with a dysbiotic gut microbiome. Additionally, an increase in proportion of HTN-associated pathogenic species of bacteria in the post-LTX microbiome will indicate a more severe occurrence of HTN. Gut-derived SCFAs exhibit protective effects against HTN development (41). This involves its ability to lower blood pressure *via* activation of G-protein coupled receptors and inhibition of histone deacetylases, according to a murine model (42). As such, reductions in SCFA-producing gut bacteria in post-LTX dysbiosis plays a role in the pathogenesis of post-LTX systemic hypertension.

CVD is also linked to a decrease in the SCFA butyrate-producing gut bacteria such as *F. prausnitzii* and *Roseburia*. This may be due to a negative correlation between the concentration of butyrate and C-reactive protein, which is recently implicated in atherosclerosis (43), the most common cause of CVD. Post-LTX dysbiosis mediated inhibition of FXR and TGR5 signaling by bile acids will aggravate atherosclerotic formation whilst inhibiting the anti-atherogenic and lipid-lowering effect of the FXR agonist INT-767 (44), thereby promoting CVD development.

Hydrogen sulfide *H*_2_*S*, which promotes vasodilation by targeting *K*_*ATP*_ channel protein, is produced *via* microbiota-driven anaerobic metabolism of sulfur-containing substrates. Liver cystathionine-γ-lyase (CSE) is involved in *H*_2_*S* generation. A deficient *H*_2_*S*/CSE system may be implicated in the development of CVD, particularly HTN due to its role in maintaining basal systolic blood pressure (45). This deficiency can be caused by dysbiosis-driven altered microbial metabolization of bile acids. This is because FXR which regulates CSE expression, is stimulated by the bile acid LCA. However, the overall extent of this impact involving the gut microbiome is not known.

The most researched microbial metabolite for CVD risk and development is Trimethylamine N-oxide (TMAO). The production of TMAO involves the gut microbial-dependent cleavage of dietary trimethylamine-containing compounds like choline to form trimethylamine (TMA), which is oxidized by liver flavin monooxygenase to form TMAO. The concentration of TMAO is influenced by changes in the composition of the gut microbiome after transplant (25). Elevated TMAO levels causes endothelial cell dysfunction through nuclear factor-κB (NF-κB) signaling. This involves the upregulation of inflammatory signals in endothelial cells, elevated oxidative stress and adhesion of leukocytes to endothelial cells, thus promoting vascular inflammation and atherosclerosis (46). TMAO also has a role in upregulating macrophage scavenger receptors, in particular scavenger receptor A1. Increased expression of these receptors promotes cellular accumulation of cholesterol *via* increased uptake of modified forms of LDLs (47). Hence, elevated TMAO levels due to a post-LTX dysbiotic microbiome can be implicated in the pathogenesis of atherosclerotic CVD and hypothesized to reflect increased risk for major adverse cardiovascular events in LTX patients.

### Post-transplant Hepatocellular Carcinoma

Resultant endotoxemia from increased bacterial translocation to the liver allograft during dysbiosis can be implicated in the pathogenesis of recurrent HCC. A mouse model simulating a human-like HCC environment through diethylnitrosamine and hepatotoxin carbon tetrachloride (*CCl*_4_) showed how heightened expression of Toll-like receptor 4 (TLR4), due to elevated LPS concentrations in the liver from increased bacterial translocation, promotes hepatocarcinogenesis in a *CCl*_4_-dependent manner. This occurs through further upregulation of the hepamitogen epiregulin by HSCs and TLR4 (48). Epiregulin acts on quiescent HSCs, which are the main progenitors for myofibroblasts in the liver. There is increased proliferation and differentiation of quiescent HSCs into myofibroblasts (49). As HCC develops, these myofibroblasts are thought to transform into cancer-associated myofibroblasts that play an important role in inducing HCC (50). Hence, it can be hypothesized that post-LTX dysbiotic microbiomes containing elevated concentrations of LPS-producing gram negative bacteria promotes the pathogenesis of HCC *via* the LPS-TLR4 pathway. Prolonged exposure of abnormal and increased LPS levels due to persistent post-LTX residual dysbiosis plays a major role in the development of HCC in the long term (48). Alterations in gut microbiota resulting in a modified BA pool also influences HCC development by affecting antitumor surveillance *via* natural killer T cells and enabling tumor cell proliferation (51). As such, an altered microbiome after LTX can play a role in increasing the risk of recurrent HCC in patients. This scenario may prompt further research post-LTX dysbiosis-related recurrent HCC and relevant therapeutic targets to prevent recurrent HCC development.

Komiyama et al. in a study investigating the role of tumor-related microbiota in the pathogenesis of *de novo* HCC, identified *Firmicutes, Proteobacteria*, and *Bacteroidetes* as dominant tumor-associated microbiota. Moreover, they discovered *Ruminococcus gnavus* as a signature microbe for hepatitis B and/or hepatitis C virus-related HCC (52). However, the mechanisms at which these microbiotas relate to HCC development is not known. Such microbial characteristics may be indicative of recurrent HCC, if they play out similarly to that of *de novo* HCC. Further development in this area could enable the use of the gut microbiome as a non-invasive biomarker for diagnosis of recurrent HCC in patients. This may also enable early detection of recurrent HCC since the presence of the tumor-associated microbial characteristics might reflect a predisposition to HCC recurrence. This is clinically relevant since detection of early HCC influences patient prognosis (53).

### Post-LTX Extrahepatic *de novo* Malignancies

LTX recipients are 2-3 times more likely to develop *de novo* malignancies as compared to the general population (54). Gut microbial dysbiosis arising from LTX contributes toward this risk of malignancy. This is because the gut microbiome influences systemic inflammation and immune homeostasis, thereby increasing host susceptibility to malignancies (55). For instance, variations in the gut microbiome can influence PD-L1, which regulates T cell response to tumor cells (56). Translocation of infectious agents, including opportunistic pathogens from the dysbiotic gut microbiome are implicated in the development of *de novo* solid organ malignancies (57). However, the specifics and mechanisms by which these microbiome-mediated malignancies occur is not clear due to limited studies.

## Discussion

The human gut microbiome plays an important role in the pathogenesis and development of complications after LTX. Hence, gut microbiota presents itself as a very useful predictive tool for post-LTX outcomes. For example, Lu et al. demonstrated how the main difference between LTX recipients and healthy controls lies in the relative abundance of butyrate-producing bacteria. Considering butyrate's capacity to modulate hepatic immunity and mediate suppression of carcinogenesis, such decreases in the gut microbiome can inform clinicians of a LTX recipient's predisposition toward disrupted allograft tolerance and even early HCC development (58). In addition, a murine model indicated the use of microbial profiling in differentiating the cause of liver dysfunction – hepatic I/R injury or ACR. Of note, the study suggested gut microbiota as a more sensitive biomarker than hepatic histology in predicting post-LTX rejection and dysfunction. As such, fecal microbiota sampling can serve as a non-invasive and potentially better biomarker for early detection or prevention of the various post-LTX complications (59).

It is highly advantageous if the presence of reliable indicators, which raise a clinician's concern of complications, increases. This assists clinicians in the deliverance of timely treatment, especially allograft rejection which necessitates prompt therapy. This manuscript has demonstrated the potential roles of the gut microbiome as these predictive and prognostic indicators for clinicians in postoperative care. For example, a clinician can conduct timely analysis of the microbiome at various stages of a patient's liver transplant process. Coupled with knowledge over the impacts of various gut microbiota, a clinician can better predict, detect and asses the risk of various complications, thereby enabling better clinical decisions and prompt treatment.

The clinical significance of the gut microbiome is compounded because of the gut-liver axis – a circular relationship occurs between hepatic disease and dysbiosis. This prompts the consideration of therapeutic alteration of gut microbiota to break this vicious cycle. In addition, given immunosuppression-mediated malignancies and the liver being an immunotolerant organ capable of supporting immunosuppression discontinuation (60), clinicians should consider therapeutic alteration of the microbiome toward a composition that prevents or improves post-LTX rejection and other complications. For example, preventing the inhibition of the antitumor immune response, which arises from constant stimulation by intestinal antigens, can be achieved *via* alteration of the gut microbiome, hence avoiding HCC progression (61). Such therapeutic alterations of the gut microbiome can be conducted through the administration of probiotics or fecal microbiota transplants (FMT). Although there is limited evidence, existing literature on probiotic usage in LTX recipients suggest some efficacy in reducing post-LTX infection rates (62). On the other hand, FMT has demonstrated to be beneficial toward alcohol-related liver diseases and hepatic encephalopathy, but no studies were found to be conducted in the context of LTX (63, 64). Nonetheless, FMT and probiotics are likely to be effective, given correlations of specific microbiota with improved LTX outcomes. More studies are required to assess their efficacy and feasibility for clinical application. An area for consideration would be the usage of probiotics and FMTs in an immunocompromised LTX recipient, which may increase risk of infectious complications.

On a separate note, immunosuppressants, which play important roles in ensuring allograft tolerance can create unforeseen alterations in the gut microbiome with plausible repercussions. For example, tacrolimus increased *Faecalibacterium prausnitzii, Bifidobacterium spp*. and decreased *Bacteroides-Prevotella, Enterobacteriaceae* (65). Of note, *Bacteroides-Prevotella* and *Enterobacteriaceae* are the main LPS-producing gram-negative bacteria. The potential reduction of endotoxins suggests an additional ability of immunosuppressants to alter microbial composition, thereby influencing the development of post-LTX complications. Hence, immunosuppressant-microbiome interactions should be another area of consideration for clinicians, particularly if unexpected outcomes occur from immunosuppressant usage in their patients.

As such, the gut microbiome should be seen as a “tool” to improve upon and aid in liver transplant patient care. However, current understandings of the gut microbiome and its alterations after LTX alongside relevant consequences is inadequate for a significant clinical application at present due to limited number of human trials and small sample sizes. Future clinical trials and studies should assess microbial alterations in LTX patients and specific microbial characteristics when considering implicated post-LTX outcomes. Large data pools are required when considering how the same microbiota may exhibit different behaviors in different contexts, where certain species could be beneficial for some but harmful for others. An example of a study that can be conducted is to investigate the microbiota promoting the pathogenesis of PTDM as it is one of the most frequent complications after LTX (66). Greater focus can also be placed into the development of more accurate and efficient forms of metatranscriptomics to greater enable clinician access to and improved incorporation of the microbiome as part of liver transplant patient care.

## Author Contributions

HW: conceptualization, data curation, writing (original draft), and writing (review and editing). WL, CN, and DT: conceptualization, data curation, and writing (review and editing). GB, AK, DH, MS, and MN: writing (review and editing) and supervision. NS and MM: conceptualization, writing (review and editing), and supervision. All authors contributed to the article and approved the submitted version.

## Funding

NS is supported by the Dean's Research Development Award (DRDA) awarded by Yong Loo Lin School of Medicine in National University of Singapore, Wong Hock Boon Society Fellowship awarded by Yong Loo Lin School of Medicine in National University of Singapore, and Junior Research Award (JRA) awarded by National University Hospital (NUH) in National University Health System, Singapore (NUHS).

## Conflict of Interest

The authors declare that the research was conducted in the absence of any commercial or financial relationships that could be construed as a potential conflict of interest.

## Publisher's Note

All claims expressed in this article are solely those of the authors and do not necessarily represent those of their affiliated organizations, or those of the publisher, the editors and the reviewers. Any product that may be evaluated in this article, or claim that may be made by its manufacturer, is not guaranteed or endorsed by the publisher.

## Abbreviations

ACR: Acute cellular rejection
BA: Bile acids
BSH: Bile salt hydrolase
CVD: Cardiovascular disease
FMT: Fecal microbiota transplant
HBV: Hepatitis B virus
HCC: Hepatocellular carcinoma
HSC: Hepatic stellate cells
HTN: Hypertension
I/R: Ischemia-reperfusion
LPS: Lipopolysaccharide
LTX: Liver transplant
SCFA: Short-chain fatty acid
TLR: Toll-like receptor
TMAO: Trimethylamine N-oxide
Tregs: Regulatory T cells.
